# A New and Improved Host-Independent Plasmid System for RK2-Based Conjugal Transfer

**DOI:** 10.1371/journal.pone.0090372

**Published:** 2014-03-03

**Authors:** Trine Aakvik Strand, Rahmi Lale, Kristin Fløgstad Degnes, Malin Lando, Svein Valla

**Affiliations:** 1 Department of Biotechnology, Norwegian University of Science and Technology, Trondheim, Norway; 2 Department of Biotechnology, SINTEF Materials and Chemistry, Trondheim, Norway; Auburn University, United States of America

## Abstract

Bacterial conjugation is a process that is mediated either by a direct cell-to-cell junction or by formation of a bridge between the cells. It is often used to transfer DNA constructs designed in *Escherichia coli* to recipient bacteria, yeast, plants and mammalian cells. Plasmids bearing the RK2/RP4 origin of transfer (*oriT*) are mostly mobilized using the *E. coli* S17-1/SM10 donor strains, in which transfer helper functions are provided from a chromosomally integrated RP4::Mu. We have observed that large plasmids were occasionally modified after conjugal transfer when using *E. coli* S17-1 as a donor. All modified plasmids had increased in size, which most probably was a result of co-transfer of DNA from the chromosomally located *oriT*. It has earlier also been demonstrated that the bacteriophage Mu is silently transferred to recipient cells by these donor strains, and both occurrences are very likely to lead to mutations within the recipient DNA. Here we report the construction of a new biological system addressing both the above mentioned problems in which the transfer helper functions are provided by a plasmid lacking a functional *oriT*. This system is compatible with all other replicons commonly used in conjugation experiments and further enables the use of diverse bacterial strains as donors. Plasmids containing large inserts were successfully conjugated and the plasmid modifications observed when *E. coli* S17-1 was used as donor were eliminated by the use of the new host-independent vector system.

## Introduction

Due to its well established genetics and good transformation competence *Escherichia coli* is the most frequently used host for manipulation of DNA via a variety of recombinant DNA technologies. After the modifications have been made it might be necessary to transfer the constructs designed in *E. coli* to alternative hosts at high frequencies. This becomes relevant for example during construction of large numbers of transposon insertion mutants or for transfer of metagenomic libraries in functional screening studies across species barriers. Transformation of naked DNA is often inefficient, or sometimes even impossible, depending on the host of interest. The use of conjugation often solves these problems as the transfer system is mainly acting in a recipient-independent manner [1]. While the recipient-independency is an attractive feature, there also exist limitations due to the requirements of complex machinery and also due to protection systems in recipient cells, such as CRISPR and restriction-modification [2], [3]. The *E. coli* strain S17-1 and its analogue SM10 are heavily used as donor strains in such transfer procedures, which is reflected by a very high citation frequency (nearly 5000 as of October 2013) of the paper in which these strains are described [4].

*E. coli* S17-1/SM10 contain a chromosomally integrated RP4 plasmid, which is essentially the same as the more studied broad-host-range self-transmissible IncP plasmid RK2 [5]. Conjugal transfer of plasmids based on this system requires the presence of an origin of transfer (*oriT*) in the plasmid to be transferred, as well as the gene products of two separate *tra*-clusters [6] which are provided *in trans* from the RP4 integrated in *E. coli* S17-1. A number of small and specialized *oriT*-containing vectors have been developed from the large RK2 plasmid (60 kb) [7], but other types of plasmids containing *oriT* may also be conjugated by *E. coli* S17-1/SM10 [8], [9].

In spite of their extensive use there are several problems associated with the *E. coli* strains S17-1 and SM10: They both contain an active bacteriophage Mu genome (within the tetracycline resistance gene of RP4) which has been shown to mobilize itself into recipient strains [10], [11]. This may cause problems as Mu DNA may randomly mutate the recipient genome and/or the transferred plasmid. Another demonstrated problem is that these strains not only mobilize *oriT*-carrying plasmids, but also their own chromosomal DNA to recipient strains at frequencies of 10 *^−^* ^4^ per donor cell [12]. Furthermore, there are also reports describing generation of plasmid modifications of unknown nature in conjugation experiments involving *E. coli* strains S17-1/SM10 [13], [14].

In addition to these findings we here report that plasmids transferred from *E. coli* S17-1 to other bacterial species quite often become modified by insertion of DNA from the donor host chromosome, presumably as a result of mobilization of DNA from the active *oriT* within the inserted RP4. This represents a rather serious problem as it very likely can lead to inactivation of genes in such transferred plasmids. There have been established alternative conjugation systems which address some of the above mentioned problems separately, such as a modified *E. coli* S17-1 strain in which the Mu genome has been inactivated [11]. In this study we present a new and improved system for conjugal transfer of mobilizable plasmids which overcomes both the problems of bacteriophage Mu and chromosomal DNA mobilization from the donor. This system is constructed in a way that all the functions required for conjugal transfer are present on a broad-host-range (RK2-compatible) plasmid, a feature that allows the use of diverse bacterial hosts as donors for conjugation of *oriT*-containing constructs.

## Materials and Methods

### Bacterial strains, plasmids and growth media

The bacterial strains and plasmids used in this study are described in Table 1. The fosmids used in the conjugation experiments are from a previous study and were constructed using the broad-host-range cloning vector pRS44 [15]. This vector harbours two replicons: *ori2,* leading to single plasmid copy in *E. coli*; and *oriV*, leading to high copy-number in strain EPI300 when induced. This strain harbours a gene encoding a high copy-number replication protein for *oriV*, TrfA, which is under a tightly regulated inducible promoter on its chromosome. Upon induction the vector copy- number increases from a single copy- to high copy-number as replication then occurs via *oriV* [16].

**Table 1:** Bacterial strains and plasmids used in this study.

Bacterial strain or plasmid	Properties^a^	Source or Reference
*E. coli*		
DH10B	F- *mcr*A Δ(*mrr-hsd*RMS-*mcr*BC) φ80*lac*Z ΔM15 Δ*lac*X74 *rec*A1 *end*A1 *ara*D139 Δ(*ara, leu*)7697 *gal*U *gal*K ë- *rps*L *nup*G	Invitrogen
EPI300	Phage T1-resistant and *lacZ^−^* strain with L-arabinose induced chromosomally expressed TrfA, ( F*^−^ mcrA Δ(mrr-hsdRMS-mcrBC) φ80dlacZΔM15 ΔlacX74 recA1 endA1 araD139 Δ(ara, leu)7697 galU galK* λ*^−^ rpsL nupG trfA tonA dhrf*)	[16]
ER2566	*F – λ– fhuA2 [lon] ompT lacZ::T7 gene1 gal sulA11 Δ(mcrCmrr) 114::IS10 R(mcr-73::miniTn10–TetS)2 R(zgb-210::Tn10 )(TetS) endA1 [dcm].* The strain is *recA+*	NEB
S17-1	*pro, res^−^ hsdR17 (rK^−^ mK^+^) recA^−^* with an integrated *RP4-2-Tc::Mu-Km::Tn7*, Tp^r^	[4]
S17-1(λ*pir)*	λ*pir* lysogen of strain S17-1	[21]
*Psedomonas. fluorescens*		
NCIMB 10525	*Pseudomonas fluorescens* wild type	NCIMB
NCIMB10525::Tn*RS48*	Derivative of NCIMB 10525 with transposon Tn*RS48* from pRS48 integrated into the chromosome	[15]
*Xanthomonas. campestris*		
B100-152	Spontaneous *xanA* exopolysaccharide-negative mutant	[22]
B100-152::Tn*RS48*	Derivative of B100-152 with transposon Tn*RS48* from pRS48 integrated into the chromosome	[15]
Plasmids		
37, 67, 83	Three different pRS44 fosmid clones carrying 35 kb inserts, Cm^r^, Km^r^	This work
pBBR1MCS-5	Cloning vector containing the broad-host-range replicon pBBR1, 4.8 kb, Gm^r^	[18]
pLITMUS28	General cloning vector, 2.8 kb, Ap^r^	NEB
pRS44	Broad-host-range combined fosmid and BAC cloning vector, 10.3 kb, Cm^r^, Km^r^	[15]
pRS48	Suicide vector with a mini-Tn*5* transposon for insertion of the *trfA* gene under *PmG5*/*xylS* control, Ap^r^, Tc^r^, 10.5 kb	[15]
pTA10	Suicide vector containing the *oriR6K* replicon and *sacB,* Cm^r^, 3.8 kb	This work
pTA15	Derivative of pTA10 containing two PCR fragments Km-1 and Km-2 (see text), Cm^r^, 5.0 kb	This work
pTA16	Derivative of pTA10 containing two PCR fragments oriT-1 and oriT-2 (see text), Cm^r^, 5.4 kb	This work
pTA17	Derivative of RK2, *oriT ^−^*, Ap^r^_,_ Km^r^, Tc^r^, 60.0 kb	This work
pTA19	Derivative of pTA17, *oriT ^−^,* Km^s^, Ap^r^_,_ Tc^r^, 59.5 kb	This work
pTA84/pTA-Mob	pTA19 derivative without the 9.4 kb AseI-AvrII fragment, containing instead a 2.8 kb pBBR1-Gm^r^ fragment, Gm^r^, 57.2 kb	This work
RK2	Ap^r^_,_ Km^r^, Tc^r^, 60.1 kb	[6]

a Ap^r^: ampicillin resistance; Cm^r^: chloramphenicol resistance; Gm^r^: gentamycin resistance; Km^r^: kanamycin resistance; Tc^r^: tetracycline resistance.

The growth media used were Lysogeny Broth (LB, 5 g yeast extract, 5 g NaCl and 10 g tryptone per litre) and Lysogeny Agar (LA, LB supplemented with 20 g agar per litre) for *E. coli* strains, LB and Difco Pseudomonas Isolation agar (PIA) for *Pseudomonas fluorescens*, and Yeasy Mold (YM) broth and YM agar for *Xanthomonas campestris*. Antibiotics were used at the following concentrations when relevant: ampicillin, 100 µg mL*^−^* ^1^ (*E. coli*); chloramphenicol, 12.5 µg mL*^−^* ^1^ (*E. coli*), 30 µg mL*^−^* ^1^ (*X. campestris*); kanamycin, 50 µg mL*^−^* ^1^ (*E. coli* and *P. fluorescens*); tetracycline 10 µg mL*^−^* ^1^ (*E. coli*), 15 µg mL*^−^* ^1^ (*X. campestris*) or 25 µg mL*^−^* ^1^ (*P. fluorescens*). Expression of *trfA* (wt, i.e. low copy-number) from *PmG5* in the recipient *P. fluorescens* and *X. campestris* strains was induced by addition of *m*-toluate at 0.5 mM, enabling replication of the transferred fosmids from *oriV*. Counter selection of *sacB* containing strains was done using sucrose at 5% (w/v). Clones in *E. coli* EPI300 were switched from single copy- to high copy-number by L-arabinose induction at 0.01% (w/v).

### Standard DNA manipulations and conjugative matings

Routine DNA manipulations and agarose gel electrophoresis were performed according to the methods of Sambrook and Russel [17], or by using commercially available kits. DNA sequencing was performed using the Big Dye Terminator version 1.1 Cycle Sequencing Kit (Applied Biosystems). Transformations of *E.coli* DH5α, ER2566 and EPI300 were performed according to the RbCl transformation protocol (New England BioLabs) or through electroporation according to Sambrook and Russel [17] (13 V cm*^−^* ^1^, 200Ω, 25 µF).

Conjugative matings were performed as follows: cells from 2 mL exponential phase growing cultures (OD ∼0.4) of donor- and recipient strains were mixed, concentrated after centrifugation and deposited onto LA without antibiotic selection (30°C, overnight). The mixtures were then plated on appropriate selective media and incubated at 30°C for 48 h, for *P. fluorescens* or 72 h for *X. campestris*.

Plasmids were isolated from cultures of *P. fluorescens* and *X. campestris* using commercial plasmid isolation kits and isopropanol precipitation, and retransformed into *E. coli* EPI300 by electroporation.

### Vector constructions

The suicide vector pTA10 was constructed through ligation of the narrow host range replicon *oriR6K*, the *sacB* gene and a chloramphenicol resistance gene, using the *pir*-expressing *E. coli* strain S17-1λpir as host. The deletions within RK2 resulting in plasmids pTA17 and pTA19 were performed through homologous recombination, using two pTA10-derivatives (pTA16 and pTA15, respectively) carrying PCR-fragments homologous to the regions flanking the deleted segments (next paragraph). The PCR-fragments within pTA15 where amplified from RK2 using the following primers (restriction sites are underlined): PCR-fragment oriT*-*1: OriT1fwdXhoI: 5'-TTTCTCGAGCCGATACGGCTCATGGATGG-3' and OriT1revEcoRI 5'-TTTGAATTCGGCAAGCGGATGGCTGATGA-3'; PCR-fragment oriT-2: OriT2fwdEcoRI: 5'-TTTGAATTCTGACGCCGTTGGATACACC-3' and OriT2revNheI 5'-TTTGCTAGCTGTCGAAGTTGC GCG AGT TA-3'. The PCR-fragments within pTA16 where amplified from RK2 using the following primers: PCR fragment Km-1: Km1fwdXhoI: 5'-TTTCTCGAGCACAAC GCCAATCAGTGATG-3' and Km1revEcoRI: 5'-TTTGAATTCTTGCTATGCAGCCGATAGAC-3'; PCR-fragment Km-2: Km2fwdEcoRI: 5'-TTTGAATTCTGCCGTGTTATGGAACTGTC-3' and Km2revNheI: 5'-TTTGCTAGCCGGTTGTCGGCAAGAACTA-3'. After amplification, the oriT-1 fragment was ligated to the oriT-2 fragment at the EcoRI sites, after which the oriT-1/oriT-2 fragment was ligated into the XhoI-NheI sites of pTA10. The procedure was repeated for the Km-1 and Km-2 PCR fragments.

### Homologous recombination for targeted deletions within RK2

For inactivation of *oriT, E. coli* ER2566 cells (*recA* ^+^) containing plasmid RK2 were first transformed with pTA16, followed by selection of chloramphenicol-resistant transformants. As pTA16 cannot replicate extra-chromosomally in this strain, these represented cells in which recombination had happened between either the *oriT*-1 or the *oriT*-2 fragment. The cells were first cultivated in the presence of chloramphenicol overnight, then, after re-inoculation (0.5% overnight-culture to fresh media), for at least 6 hours in the absence of selection. Different dilutions were next plated on LA plates containing sucrose, selecting for cells not containing *sacB*, i.e. where also the second cross-over had occurred (at the remaining *oriT* fragment). Correct alteration was confirmed through PCR reactions and sequencing, and in addition the new RK2 derivative (pTA17) was confirmed being conjugation deficient (due to inactivation of *oriT*).

Construction of the kanamycin sensitive derivative of pTA17 (pTA19) was performed similarly as described for pTA17, but with the use of pTA15 instead of pTA16.

### Construction of pTA-Mob

The mobilization plasmid pTA-Mob was constructed from pTA19 by first removing the AseI-AvrII fragment (9.4 kb) containing the replication origin, *oriV*, and the ampicillin and tetracycline resistance genes. An AseI-AvrII fragment containing the broad-host-range replicon pBBR1, as well as a gentamycin resistance gene was next ligated into the same restriction sites. This pBBR1rep-Gm^r^ fragment was amplified from plasmid pBBR1MCS-5 [18] in two PCR reactions using the following primer pairs (restriction sites are underlined): Gent-Fwd: 5'-CGTATTGCATTAATCCACCTGGCGGCGTTGTGAC-3' and Gent-Rev: 5'-CGAATTCCTGCCGACATGGAAGCCATC-3', pBBR1-Fwd: 5'-CGAATTCATACCCACCGGCTCCAACTG-3' and pBBR1-Rev: 5'- TCCTAGGTTAAACGCCTGGTGCTACGC 3'.

## Results and Discussions

### Detection of plasmid-modifications after conjugal transfer from *E. coli* strain S17-1 to *Pseudomonas fluorescens* and *Xanthomonas campestris*

**Figure 1:**
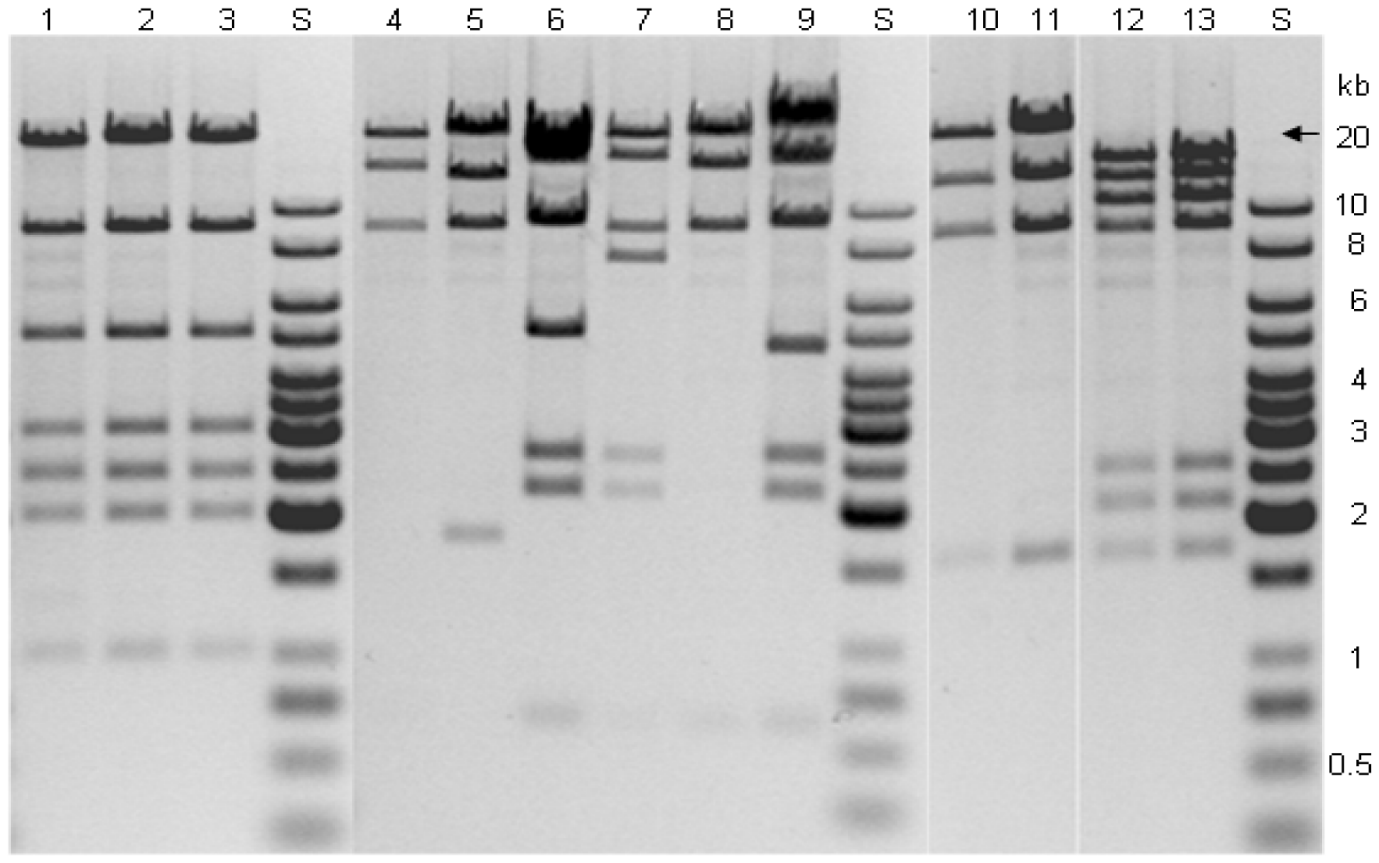
Agarose gel electrophoresis analysis of HindIII-digested fosmid clones before and after conjugation from *E. coli* S17-1 to *P. fluorescens::*Tn*RS48* or *X. campestris::*Tn*RS48*. Lane 1, plasmid 62 before transfer, and lanes 2 and 3 after transformation to *E. coli* from *P. fluorescens* and *X. campestris*, respectively. Lane 4, plasmid 83 before conjugal transfer, and after transformation to *E. coli* from *P. fluorescens* (lanes 5-7) and *X. campestris* (lanes 8 and 9). Lane 10, plasmid 37 before transfer, and after transformation to *E. coli* from *P. fluorescens*. (lanes 11 and 12) and *X. campestris* (lane 13). S: Molecular weight standard (Fermentas).

In an extension of the previously reported inter-species transfer experiments with fosmids from a metagenomic library [15], we discovered that some of the fosmids had increased in size after being conjugally transferred to *P. fluorescens* and *X. campestris* (see *Materials and Methods* for information about the fosmids). For analyses of the plasmids within transconjugants of *P. fluorescens* and *X. campestris*, the plasmids needed first to be retransformed into *E. coli* EPI300 in order to obtain high quality plasmid DNA. This step also ensures that the analysis is performed on only one fosmid. Fosmid preparations of the transferred/retransformed fosmids were then digested with restriction endonuclease HindIII and the resulting fragments were separated by agarose gel electrophoresis. The digestion patterns were then compared to the corresponding results obtained from the original plasmid preparations from the *E. coli* donor strains (Figure 1). Lanes 1-3 show a case where the restriction fragment band patterns for a randomly selected plasmid, designated 62, remain the same after conjugal transfer both to *P. fluorescens* and *X. campestris*. However, for another plasmid, designated 83, the restriction fragment band patterns were altered after being conjugated to *P. fluorescens* (lanes 5-7) (original fosmid band pattern in lane 4). Of the plasmids obtained after conjugation of fosmid 83 to *X. campestris,* one appeared very similar to the original fosmid in the *E. coli* donor (lane 8), while the band pattern originating from another *X. campestris* transconjugant was clearly different (lane 9). A final example is illustrated by transfer of a fosmid designated 37 (lane 10 shows the original fosmid). No obvious differences could be observed for the fosmid after transfer to *P. fluorescens* (lane 11), but the fosmids in lanes 12 (from *P. fluorescens*) and 13 (from *X. campestris*) were clearly different from the one in the donor. Notably, these two lanes display indistinguishable restriction band patterns even though the plasmids had been transferred to two different species, indicating that the modifications were originating from the donor.

The experiments described above revealed that fosmid modifications occasionally occur for a given fosmid, that each individual transfer event can lead to a different outcome for a given fosmid, that passage through two different hosts can apparently lead to the same modification, and finally that the modifications always involve increase in the fosmid size.

### Analysis of the inserted DNA

To characterize the nature of the apparently inserted DNA, ten such altered DNA bands were excised from the agarose gel, purified, ligated into pLitmus28 and end-sequenced. Three of the obtained ten sequences did not give any significant hits against the public databases when BLAST analysis was performed, indicating that these were originating from the metagenomic insert DNA. Analyses of the remaining seven sequences revealed that four of these originated from the *E. coli* donor strain chromosome, all within a region of ca. 35 kb, whereas the last three fragments were from a gene within the Tn7 transposon. Historically the Tn7 was used to inactivate the kanamycin resistance gene within RP4 during construction of the conjugation *E. coli* donor strain S17-1 [4].

Based on these findings it appeared that the fosmid modifications were results of insertions of chromosomal DNA from the donor into the fosmids. We suspected that the inserted chromosomal DNA was located near the integrated RP4, and that the insertions do occur as a consequence of co-activation of the chromosomally located *oriT* element (within RP4) during conjugation. As the fosmid vector used (pRS44) contains several elements from the RK2 plasmid, including *oriV, oriT* and *parDE,* it seemed possible that homologous recombination may occur between fosmid- and RP4 sequences. Correspondingly, the Tn7-sequences could possibly originate from the Tn7-transposon inserted into the kanamycin resistance gene of the integrated RP4, alternatively partly due to Tn7 transposition. As *E. coli* S17-1 is *recA^−^*, homologous recombination should not occur within this donor host. However, it appeared fully feasible that the fosmid and DNA mobilized from the chromosomally located *oriT* could be co-transferred to the same recipient cell, and that homologous recombination could occur between these single-stranded molecules, given that the recipient was *recA* ^+^. Such a hypothesis may explain how DNA fragments originating from the *E. coli* chromosome are integrated into the fosmid clones.

### Construction of a new mobilization system for transfer of any plasmid containing oriT

Given that the observed modification problems occur as a result of DNA-mobilization from *oriT* in the *E. coli* S17-1 chromosome, inactivation of *oriT* within the donor strain should eliminate the problem as this would block mobilization of the chromosomal DNA (a view also described by Babic and co-authors [12]). However, we also sought to eliminate the possibility of Mu genome mobilization as well as other reported *E. coli* S17-1/SM10-related problems, and in addition we wanted to circumvent the restriction of using one particular strain as donor. A plasmid-based mobilization system that provides the *tra*-gene products for mobilization of *oriT*_RK2_-containing vectors, which itself does not contain an intact *oriT,* could solve these issues. The most obvious strategy to achieve this appeared to be the substitution of the replication functions in an *oriT*-inactivated version of RK2 with another replication system. To avoid incompatibility problems with the plasmids to be transferred, the replicon of this new plasmid should satisfy three criteria: (i) it should not belong to the incompatibility groups of those plasmids that are heavily used in conjugal gene transfer experiments in bacteria, (ii) it should stably maintain the large regions from RK2 necessary to ensure intact Tra functions, and (iii) the new replicon should also be able to replicate in various bacteria (pBBR1 based plasmids are known to replicate in *Alcaligenes eutrophus, Bartonella bacilliformis, Bordetella spp., Brucella spp., Caulobacter crescentus, E. coli, Gluconacetobacter xylinus, Paracoccous denitrificans, Pseudomonas fluorescens, P. putida, Rhizobium meliloti, R. leguminosarum by. viciae, Rhodobacter sphaeroides, Salmonella typhimurium, Vibrio cholerae, X. campestris*). For this purpose the pBBR1 replicon was chosen which satisfies all the criteria listed above [18], [19].

**Figure 2:**
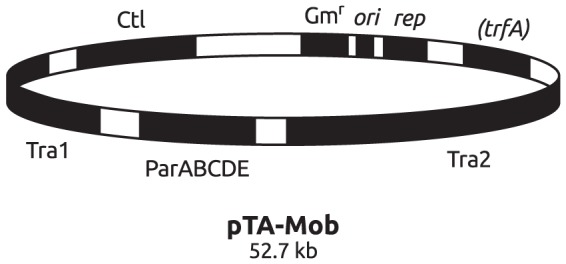
Map of the mobilization helper plasmid pTA-Mob with relevant regions depicted. Gm^r^, gentamycin resistance gene; *rep*, pBBR1 replication protein gene; *ori*; pBBR1 replication origin; (*trfA*), replication initiation protein gene from the RK2 replicon, this replicon is not active due to lack of RK2 replication origin *oriV*; Tra1 and Tra2, regions containing the *tra* genes necessary for conjugative transfer of *oriT* containing plasmids; *parABCDE*, stabilization region encoding the gene products ParA, B, C, D and E; Ctl, central control operon of RK2 [6].

In order to make precise modifications of the large-sized RK2 plasmid (60 kb), a system was developed that allows for homologous recombination between ligated fragments within a suicide plasmid (pTA10) and the target region of RK2, with subsequent selection of the altered RK2-derivatives (see *Materials and Methods*). This system was used to make two deletions within the RK2 plasmid, resulting in inactivation of the *oriT* site as well as the kanamycin resistance gene, generating the plasmid pTA19. Further, a fragment in pTA19 containing the replication origin, *oriV,* and the ampicillin and tetracycline resistance genes was replaced with a fragment containing the pBBR1 replicon and the gentamycin resistance gene (giving pTA-Mob, Figure 2).

**Figure 3:**
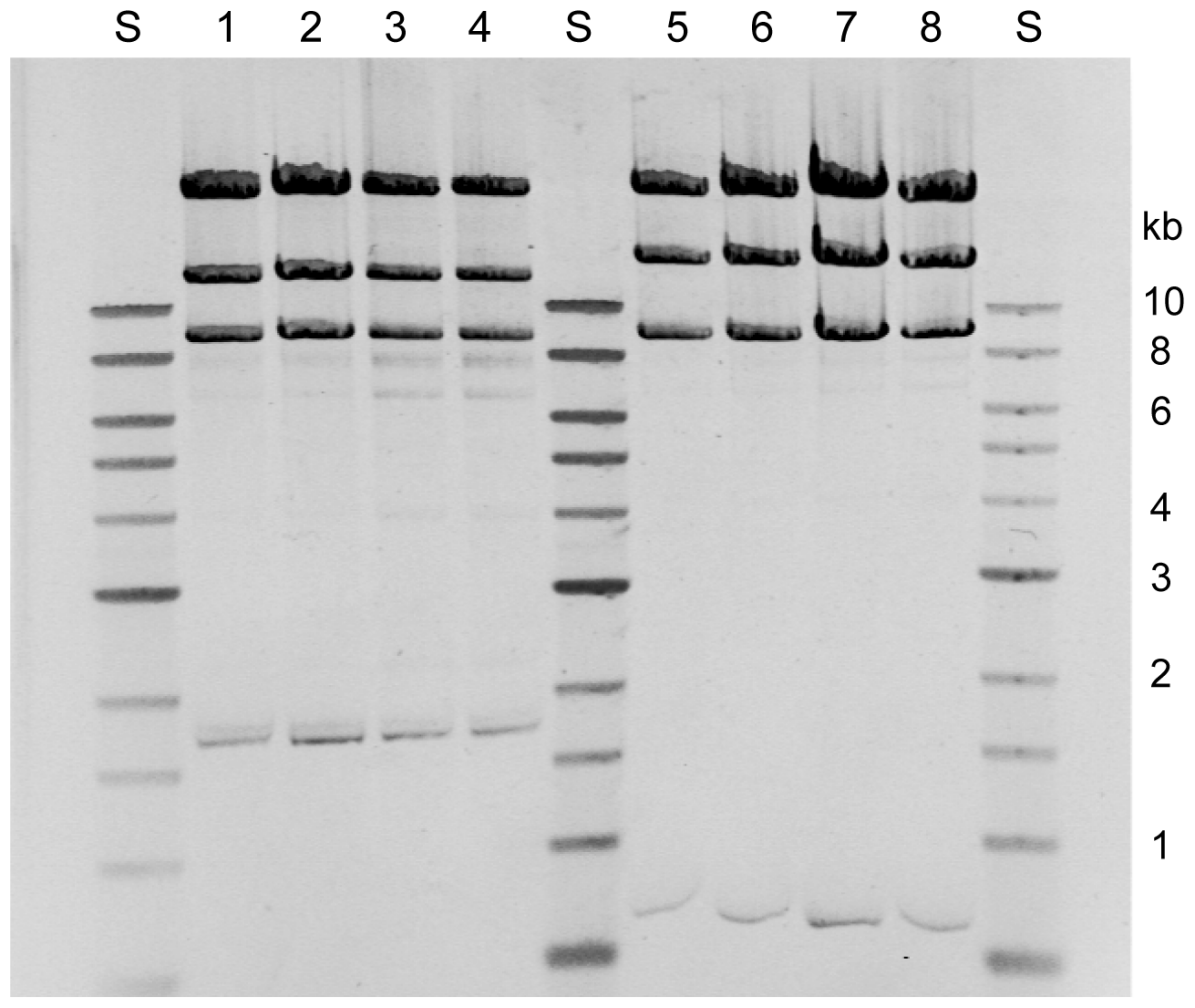
Agarose gel electrophoresis analysis of HindIII-digested fosmid clones that have been conjugatively transferred from *E. coli* DH10B/pTA-Mob to *P. fluorescens::*Tn*RS48*. Lane 1: plasmid 37 before transfer and lanes 2-4 after conjugation. Lane 5: Plasmid 83 before transfer and lanes 6–8 after conjugation. S: Molecular weight standard (NEB).

Plasmids up to 220 kb in size were successfully transformed into *E. coli* DH10B/pTA-Mob cells, demonstrating that very large plasmids are maintained together with the relatively large-sized pTA-Mob. Next, *oriT*-containing plasmids were conjugated from *E. coli* DH10B/pTA-Mob cells using *P. fluorescens* as recipient, and the relatively easy attainment of transconjugants confirmed that the RK2 Tra functions were still intact. To test the system with respect to the described problem concerning modifications of conjugatively transferred plasmids, fosmid clones 37 and 83 were analysed after mobilization from *E. coli* DH10B/pTA-Mob. Plasmids from 11 different *P. fluorescens* transconjugants were digested with HindIII as described above (Figure 1), and the DNA fragments generated were analysed by agarose gel electrophoresis analysis (Figure 3). As it can be seen, the restriction band patterns of plasmids originating from transfer experiments with fosmids 37 (three examples are given in lanes 2–4) and 83 (lanes 6–8) were all similar to that of the original fosmids (lanes 1 and 5).

Based on these findings we conclude that the established broad-host-range mobilization plasmid pTA-Mob is suitable for supporting *in trans* the necessary *tra*-genes for conjugation of *oriT*-containing plasmids from presumably a large number of bacterial species, and that its use also leads to elimination of all known problems associated with the heavily used *E. coli* S17-1/SM10 strains. Since pTA-Mob is a broad-host-range plasmid, non-*E. coli* hosts may also potentially be used as donors for conjugation, given that the plasmid can replicate in these host(s) [18]. We have for instance previously shown that conjugation from *E. coli* is inefficient at low temperatures [20], indicating that it might be difficult to conjugate plasmids from *E. coli* to strictly psychrophilic species. With this new mobilization system this can potentially be solved through the use of a pTA-Mob containing “intermediate host” which handles the temperature-requirements of both strains. Such a strategy could also be used in any case where mixing of the host of interest with *E. coli* cells causes some form of problem. We also envision that the new system might be used as a model in environmental microbiology studies involving gene transfer between various genus/species.
